# Dietary Intervention and DEHP Reduction: Rudel et al. Respond

**DOI:** 10.1289/ehp.1103852R

**Published:** 2011-09-01

**Authors:** Ruthann Rudel, Janet Ackerman, Robin Dodson

**Affiliations:** Silent Spring Institute, Newton, Massachusetts, E-mail: rudel@silentspring.org

Steven Risotto, representing phthalate manufacturers for the American Chemistry Council (ACC), commented on our study that found a 3-day diet with limited food packaging reduced participants’ average bis(2-ethylhexyl) phthalate (DEHP) exposure by > 50% (Rudel et al. 2011).

Risotto’s statement that creatinine adjustment by normalization is accepted practice is misleading. Creatinine normalization is appropriate in a longitudinal study if the daily creatinine excretion of the participants remains approximately constant. That assumption is not reasonable in a dietary intervention because short-term changes in diet can strongly influence creatinine levels (Kesteloot and Joossens 1993). In our article (Rudel et al. 2011), we addressed urinary dilution by including creatinine as a variable in the mixed-effects model that estimates exposure reduction from the intervention, as currently recommended by researchers at the Centers for Disease Control and Prevention (Barr et al. 2005). Our analysis showed significant decreases of 53–56% in the three DEHP metabolites. Because creatinine normalization is common, we also included normalized results. Creatinine levels dropped significantly during the intervention, indicating that creatinine normalization artificially reduced the observed change. Nonetheless, results showed a 42–45% decrease in all three DEHP metabolites; the decrease was statistically significant for the most abundant metabolite, MEHHP (mono-(2-ethyl-5-hydroxyhexyl) phthalate).

Risotto also questions whether DEHP reductions are attributable to two individuals with high initial exposures. However, we reported the decreases in geometric means, which are not strongly influenced by a few high values. After removing these two participants, we still observed decreases of 37–42% in the geometric means of DEHP metabolites, and reductions in the two most abundant metabolites remain statistically significant. Removing participants with high preintervention exposures is appropriate if an unknown exposure may have covaried with the intervention, but because the two highest exposures were in different families, such confounding seems unlikely.

As to why DEHP metabolite levels dropped during the intervention but did not increase significantly after the intervention—as discussed in detail in our article (Rudel et al. 2011)—the discrepancy may be attributable to the different-length “washout periods” (~ 48 hr between the beginning of the intervention and the first intervention urine sample, and ~ 36 hr between when participants resumed their regular diet and the first postintervention urine sample).

Risotto questions the public health significance of our observed reduction in DEHP exposure. However, DEHP exposure levels in our study (Rudel et al. 2011)—and in the U.S. population—are similar to or higher than those recently reported to exceed health guidelines. Koch et al. (2011) found that 5 of 108 children studied had daily DEHP intakes in excess of the current U.S. Environmental Protection Agency reference dose, and 25% exceeded the tolerable daily intake for cumulative exposure to several antiandrogenic phthalates that act additively. This risk assessment contradicts the misleading ACC press release about our study, which claimed “consumer exposure to BPA [bisphenol A] and DEHP, from all sources, is up to 1,000 times lower than government-established safe exposure levels” (ACC 2011). This statement suggested to reporters that consumer exposures are much lower than government health guidelines, when in fact a substantial percentage of DEHP exposures are above guidelines.

Although our findings (Rudel et al. 2011) indicate that food packaging was a major source of DEHP in early 2010, we are encouraged by Risotto’s news that the industry believes DEHP is no longer used in food packaging. If manufacturers provided comprehensive information about chemicals in products, scientists, regulators, and consumers would not have to resort to expensive studies to understand potential risks and opportunities for mitigation. Not knowing the new packaging formulations, we cannot evaluate them. We hope manufacturers have carefully tested the DEHP substitutes for endocrine disruption and other safety concerns.
